# How healthcare professionals respond to parents with religious objections to vaccination: a qualitative study

**DOI:** 10.1186/1472-6963-12-231

**Published:** 2012-08-01

**Authors:** Wilhelmina LM Ruijs, Jeannine LA Hautvast, Giovanna van IJzendoorn, Wilke JC van Ansem, Glyn Elwyn, Koos van der Velden, Marlies EJL Hulscher

**Affiliations:** 1 Academic Collaborative Centre AMPHI, Dpt of Primary and Community Care, Radboud University Nijmegen Medical Centre, Geert Grooteplein 21, 6525, EZ Nijmegen, The Netherlands; 2 Municipal Health Service GGD Rivierenland, J.S. de Jongplein 2, 4001, WG Tiel, The Netherlands; 3 Dpt of Primary Care and Public Health, Cardiff University School of Medicine, Neuad Meironnydd, Heath Park, Cardiff, CF, 14 4YS, United Kingdom; 4 Scientific Institute for Quality of Healthcare, Radboud University Nijmegen Medical Centre, Geert Grooteplein 21, 6525EZ, Nijmegen, The Netherlands

**Keywords:** Vaccination, Immunization, Healthcare professionals, Decision-making, Religion, Religious objections to vaccination

## Abstract

**Background:**

In recent years healthcare professionals have faced increasing concerns about the value of childhood vaccination and many find it difficult to deal with parents who object to vaccination. In general, healthcare professionals are advised to listen respectfully to the objections of parents, provide honest information, and attempt to correct any misperceptions regarding vaccination. Religious objections are one of the possible reasons for refusing vaccination. Although religious objections have a long history, little is known about the way healthcare professionals deal with these specific objections. The aim of this study is to gain insight into the responding of healthcare professionals to parents with religious objections to the vaccination of their children.

**Methods:**

A qualitative interview study was conducted with health care professionals (HCPs) in the Netherlands who had ample experience with religious objections to vaccination. Purposeful sampling was applied in order to include HCPs with different professional and religious backgrounds. Data saturation was reached after 22 interviews, with 7 child health clinic doctors, 5 child health clinic nurses and 10 general practitioners. The interviews were thematically analyzed. Two analysts coded, reviewed, discussed, and refined the coding of the transcripts until consensus was reached. Emerging concepts were assessed using the constant comparative method from grounded theory.

**Results:**

Three manners of responding to religious objections to vaccination were identified: providing medical information, discussion of the decision-making process, and adoption of an authoritarian stance. All of the HCPs provided the parents with medical information. In addition, some HCPs discussed the decision-making process. They verified how the decision was made and if possible consequences were realized. Sometimes they also discussed religious considerations. Whether the decision-making process was discussed depended on the willingness of the parents to engage in such a discussion and on the religious background, attitudes, and communication skills of the HCPs. Only in cases of tetanus post-exposure-prophylaxis, general practitioners reported adoption of an authoritarian stance.

**Conclusion:**

Given that the provision of medical information is generally not decisive for parents with religious objections to vaccination, we recommend HCPs to discuss the vaccination decision-making process, rather than to provide them with extra medical information.

## Background

Vaccination programs have successfully controlled many infectious diseases. In recent years, however, healthcare professionals (HCPs) have faced increasing concerns about the value of childhood vaccination [1]. Parental decision making with regard to vaccination is complex. Medical, psychological, social, and cultural aspects can play a role [2-4]. Moreover, the medical information provided and trust in the HCP can play a role as well [5-7].

Although not all HCPs recommend childhood vaccinations according to the national immunization schedule [8,9], most are convinced of the value of vaccination and many find it difficult to deal with parents who object to vaccination.

Religious objections are one of the possible reasons for refusing vaccination. In the Netherlands, an orthodox Protestant minority of about 250,000 members has religious objections to vaccination. Forty percent of them has been found to *not* be vaccinated at all [10]. Epidemics of polio, measles, rubella, and mumps have broken out among this group and spread to their relatives in Canada [11-14]. Orthodox Protestant objections to vaccination focus on the necessity of trust in divine providence. On biblical grounds arguments for vaccination are put forward as well: vaccination may be considered as a gift of God to be used in gratitude [15]. Orthodox Protestant churches leave it up to parents to decide to have their children vaccinated or not.

During the polio epidemic of 1978, Veenman and Jansma identified among orthodox Protestants religious objections, family tradition, and fear of possible side-effects as major reasons for not being vaccinated [16]. More recently, we performed a study on vaccination decision-making among orthodox Protestant parents and found that vaccinating as well as non-vaccinating parents predominantly used religious arguments to justify their decision. If side-effects of vaccination were mentioned, they often had a religious connotation. Non-vaccinating parents who primarily refused vaccination because of interference with divine providence, also mentioned that man is not allowed to cause disease in a by God given healthy body. On the other hand orthodox Protestant parents who broke with tradition and participated in the National Immunization Program (NIP), interpreted side-effects as a sign of God that they had made the wrong choice [17].

In the Netherlands, all children are offered vaccination free of charge via local child health clinics (CHCs) as part of a NIP (see Table 1). CHC staff consists of trained CHC doctors and trained CHC nurses. They also monitor the children’s growth and development [18]. During the standard home visit for every newborn baby, the CHC nurse provides the parents with vaccination information and registers whether the child will participate in the NIP or not. If the parents are unsure, the topic is addressed by the CHC doctor during the first consultation at the CHC. Participation in the NIP is on voluntary basis; vaccination coverage is nevertheless high: more than 95% in 2-year olds [19]. 

**Table 1:** National immunization schedule in The Netherlands

**Phase**	**Age**	**Injection 1**	**Injection 2**
1	0 months	HBV*	
	2 months	DTaP-IPV/Hib/HBV	Pneu
	3 months	DTaP-IPV/Hib/HBV	Pneu
	4 months	DTaP-IPV/Hib/HBV	Pneu
	11 months	DTaP-IPV/Hib/HBV	Pneu
	14 months	MMR	MenC
**2**	4 years	DTaP-IPV	
**3**	9 years	DT-IPV	MMR
**4**	12 years	HPV**	

* Only for children of a mother who tested positive for hepatitis B.

** Only for girls: Three injections with a one-month interval between the first and second and a five-month interval between second and third.

HBV = Hepatitis B.

DTaP-IPV/HIb/HBV = Diphtheria Tetanus acellular Pertussis Inactivated Polio vaccine/Haemophilus Influenzae type B/Hepatitis B.

Pneu = Pneumococci (tenvalent).

MMR = Measles Mumps Rubella.

MenC = Meningococci C.

HPV = Human Papilloma Virus.

General practitioners (GPs) or family physicians are not involved in the NIP. Other medical care, however, primarily involves GPs. People are listed with a GP who provides general medical care and who coordinates access to specialists and hospital care [20]. The GPs conduct an Influenza Immunization Program, focused on adults and children with a medical indication such as chronic heart or lung disease. Like the NIP this Influenza Immunization Program is offered free of charge.

Religious objections to vaccination have a long history, nevertheless little is known about the way HCPs deal with these specific objections. The American Academy of Pediatrics issued a guideline “Responding to parental refusals of immunization of children” which advises to listen respectfully to all objections, provide honest information, and attempt to correct any misperceptions [21]. A Dutch brochure on objections to vaccination advises largely the same [22]. Few papers were published on the actual response of health care professionals to parents with objections to vaccination [23,24]. The objections in these studies concerned vaccine safety and HCPs responded to them by trying to convince the parents of the medical benefits of vaccination. The response of health care professionals to parents with religious objections to vaccination has -to our knowledge- never been studied.

A qualitative study was therefore undertaken to gain insight into the responses of HCPs to parents with these specific objections to the vaccination of their children. The research questions were:

How do HCPs respond to parents with religious objections to vaccination?

Which determinants influence HCPs’ responses to parents with religious objections to vaccination?

## Methods

### Study design

Because of the explorative character of our study we chose a qualitative research design using a grounded theory approach [25]. Semi-structured interviews were undertaken with HCPs who had ample experience with orthodox Protestant parents. According to the grounded theory approach, inclusion of participants was continued until data saturation was reached, coding of the data was entirely based on their content, and emerging concepts were assessed for consistency by reviewing previously coded interviews.

### Study population

The study population was composed via purposeful sampling. Participants were recruited in villages with large orthodox Protestant populations. The selection of these villages was based on the results of a previous study [26]. HCPs with different professional backgrounds (CHC doctors, CHC nurses and GPs) and different religious backgrounds were approached by the researchers and invited to participate. HCPs who had little or no experience with orthodox Protestants were excluded. Inclusion of participants was continued until data saturation was reached; this was after 22 interviews. We initially approached 6 more HCPs, 5 of them were excluded because they were not seeing orthodox Protestant patients and 1 HCP could not be interviewed due to logistic problems. Thus, in the end, 22 HCPs who all had ample experience with religious objections to vaccination were interviewed: 7 CHC doctors, 5 CHC nurses and 10 GPs. Six of them were members of orthodox Protestant churches. Participant characteristics are summarized in Table 2. 

**Table 2:** Characteristics of participants

	**CHC doctors**	**CHC nurses**	**GPs**
**N**	7	5	10
***Gender***			
Male	0	0	10
Female	7	5	0
***Religion***			
Orthodox Protestant	1	0	5
Protestant	3	1	3
Other or no religion	3	4	2
***Working experience***			
Mean (years)	18	8	24
Range(years)	4-29	1-17	4-32

### Data collection

Two interviewers (GvIJ and WLMR) visited the HCPs between January 2009 and June 2010 at their practices to interview them with regard to a number of vaccination topics (see Table 3). The topic list was constructed on the basis of an exploratory meeting with key persons from the orthodox Protestant community, the NIP and CHCs who were represented in the advisory committee of the project. The interviews lasted an average of 30 minutes (range 20–45 minutes). Because vaccination is a particularly sensitive subject among orthodox Protestant parents, observation of consultations was not feasible.

**Table 3:** Interview topics

	**Main topic**	**Additional questions**
**1**	Spontaneous questions or remarks of orthodox Protestant parents on the topic of vaccination	- On medical aspects / adverse reactions / religious aspects / catch up vaccinations / regrets following vaccination / other aspects
		- Response to these questions
**2**	Raising the topic of vaccination during consultations	- When, why and how?
**3**	Insight into parental decision making	- Time of decision making and reconsideration:
		newborns /epidemics
		- Specific circumstances:
		travel / work / wounds (tetanus)
		- Decision-making process:
		influence of partner /family /friends
		influence of clergymen and church members
		- Decisive factors in decision-making
**4**	Experience working in communities with low vaccination coverage	
**5**	Affinity with orthodox Protestant religion	

### Analysis

The interviews were recorded and transcribed verbatim. The transcripts were thematically analyzed using the qualitative software program Atlas.ti 6.0. Two analysts (WvA and WLMR) independently coded the transcripts and subsequently reviewed, discussed, and refined the coding schemes until consensus was reached. All transcripts were coded and discussed by both analysts. Emerging concepts were assessed using the constant comparative method from grounded theory: previously analyzed interviews were reviewed in order to check if their content fitted into the concept [25].

## Results

HCPs reported three different manners of responding to religious objections to vaccination: provision of medical information, discussion of the vaccination decision-making process, and adoption of an authoritarian stance. These manners of responding are described in greater detail below. The manner of responding which was applied depended on characteristics of the child, the child’s parents, and the HCP him/herself. For each manner of responding the determinants are described.

### Provision of medical information

All HCPs reported to respond to religious objections to vaccination predominantly with medical information. They stated that the provision of medical information was their most important contribution to vaccination decision-making, nevertheless many of them thought the provision of such information to be not very rewarding.

Respondent 2, CHC doctor

They think measles is not that serious, it’s just a childhood disease . But measles can be really serious and I try to explain that, that it may have serious complications.

Respondent 5, CHC doctor

There is still some ignorance. How vaccination works, that it doesn’t cause disease, the side-effects. I’ll explain that.

Respondent 12, CHC nurse

They’re not impressed by mumps. And whooping cough? I explain that infants may even die of lack of breath, that’s the risk if they’re not vaccinated. But that doesn’t result in enough fear to make them start vaccination, even not in the presence of whooping cough at school. They just wait and see.

Respondent 15, GP

I told them about the immune system and antibodies. How that has been created in the human body, and what vaccination exactly does.

Respondent 10, CHC nurse

You may give them a lot of information, tell them that it is better to vaccinate, but they do not change their point of view.

Respondent 2, CHC doctor

It remains hard. I regularly tell them what the illnesses do and also refer them to our website. On the basis of that information, very few come around to vaccination, however. And then you lose heart.

CHC doctors and nurses reported to provide information on the severity of the vaccine preventable diseases, the benefits of vaccination, and its possible side-effects. They said they attempted to correct any misperceptions and offered later vaccination to parents who were in doubt. The extent of the information that was provided was determined by a characteristic of the child: being firstborn or not. When the child was not a firstborn, most CHC doctors and nurses simply asked if the parents still objected to vaccination. Orthodox Protestant families are large, and CHC doctors and nurses reported not wanting to “bother” the parents too much for fear that they would stop coming to the CHCs for monitoring.

The same was found for the GPs offering influenza vaccination. All of the GPs provided patients with medical information about the vaccination. Moreover, the specific risks of influenza for the patient with a particular medical condition were explained. After repeated vaccination refusal, the GPs generally reported stopping discussion with the patients.

### Discussion of the decision-making process

In addition to the provision of medical information, the HCPs sometimes discussed the vaccination decision-making process itself. That is, the HCPs verified just how the decision not to vaccinate was made and whether or not the possible consequences of non-vaccination were realized. Some HCPs said they also briefly discussed religious considerations, while others suggested the parents to read a booklet on the religious arguments for and against vaccination published by some orthodox Protestant ministers [15]. Still others systematically discussed all religious arguments for and against vaccination. 

Respondent 8, CHC nurse

If they are in doubt I’ll discuss that. But I cannot advise them in religious matters. I ask them what they want to know and send them some information brochures. And we note in the case history at the subject vaccination “in doubt”. So next time the doctor will come back to it.

Respondent 1, CHC doctor

I stress that it’s a personal decision. It’s all right with me if they don’t vaccinate, as long as it is a deliberate choice. I always ask them if they know any people from their church who do vaccinate. They never know. And then I tell them that more and more people, also from their church, choose to vaccinate. I try to entice them away from tradition.

Respondent 3, CHC doctor

[I discuss with them] how they reached their decision, if they have talked about it with others, or if they agree. And if they are aware of the possible consequences. Can they live with that? If your child becomes afflicted, will your faith be enough? Will you be able to handle things, accept things, not have a moral dilemma. Parents who can respond to all of this, they have carefully thought things through.

Whether HCP s discussed the vaccination decision-making process or not depended first and foremost upon the willingness of the parents to engage in such a discussion.

In addition, discussion of the decision-making process depended on HCP-related factors: their religious backgrounds, their attitude to religious objections to vaccination and their communication skills, see Table 4.

**Table 4:** Determinants of the professional influencing the discussion of the decision-making process

	**Determinant**	**Quotes**
**1**	**Religion**	
**1a**	**Orthodox Protestant**	*Especially when I show them that I know these denominations, they tell more about their deliberations. I think they tell me a lot more than most of my colleagues. (R5, CHC doctor)*
		*They know that I’m a confessor of one of the orthodox Protestant denominations, therefore they come to me with their questions. I can easily go into it, because I feel what the problem is.(R18, GP)*
**1b**	**Protestant**	*They are not talking to someone who knows nothing about it. They have the idea that I can place myself in their shoes and know the terminology. (R1, CHC doctor)*
		*I show them that I’m interested in their background, and I tell them about my Protestant background. Not orthodox, but just Protestant. That makes a difference, they expect that I will understand them. And that’s why they tell me about their considerations. (R4, CHC doctor)*
**1 c**	**Other or no religion**	*Sometimes I ask them: “What is it, that is written in the Bible?” And then I get a phrase that I don’t understand at all. (R12, CHC nurse)*
**2**	**Attitude towards parents with religious objections**	*I don’t have any affinity with their religion. At that moment [during the polio-epidemic] I couldn’t imagine that you refused to have your children vaccinated. I rather got angry than that I tried to understand it. I still don’t understand it, or maybe I don’t want to understand it, that’s also possible. (R17, GP)*
		*The moral dilemma, I can’t relate to that. It is something that doesn’t play a role on my part at all….I can only indicate what we vaccinate for; they have to fight the moral battle themselves. (R8,CHC nurse)*
		*There are always people who don’t accept it. That’s their philosophy of life, and I resigned to it, through the years. It’s their way of thinking and you have to respect it. (R 21, GP)*
		*I find it interesting to learn about their arguments, to talk about it. (R1, CHC doctor)*
		*My approach is to go along with them, I know why they didn’t have their children vaccinated, at least I think I know, and from that point of view I reason why this specific vaccination would be necessary, or not.(R14, GP)*
**3**	**Communication skills**	*This isn’t part of providing sound medical information […]You certainly feel that you would like to do something more, but you don’t know what form to give this. (R6, CHC doctor)*
		*I’m glad if I am able to discuss the subject and get to know why parents decide to vaccinate their children. But I don’t find out why they refuse. That is more difficult. (R4, CHC doctor)*

Especially an orthodox Protestant background, or at least proper knowledge of the orthodox Protestant religion, was reported to be important. The orthodox Protestant GPs reported being consulted — a few times a year —by orthodox Protestant parents for advice on whether to have their children vaccinated or not. In these cases, most of the GPs reported systematically discussing both the medical and religious arguments for and against vaccination with the parents.

Respondent 14, GP

I try to find out how they feel about vaccination and why they came to me to talk about it. Apparently they’re not sure what to do. They like to hear the arguments for and against, and I know the medical arguments. But I also know the religious arguments and these arguments are discussed as well. In fact, it’s more pastoral than medical.

Respondent 16, GP

They sometimes ask: “What should I do?” That’s difficult, I don’t answer such a question. They have to decide themselves. I give them some material, on which they can base their choice. I show them the pros and cons, medically but also religiously. In the Bible there are arguments for and against vaccination, but it’s up to them to weigh these arguments.

Respondent 22, GP

I try to tell the whole story, objectively. About the smallpox vaccination in the past and modern vaccines today. I also mention that it’s important that they can account for their choice. If you don’t feel right about it, you should ask yourself if you should continue in that direction or reconsider your choice.

One of the orthodox Protestant GPs even reported using religious considerations to support the final decision making in a dissenting couple.

Respondent 14, GP

If one does and the other doesn’t, then I point to their vows; they are married; they promised in the church that the wife would follow the husband.... If they cannot figure things out themselves, then the husband as head of the family should make the decision and the wife follow him on this. This sometimes works.

Although all orthodox Protestant GPs realized that they could set an example, none of them revealed the vaccination status of their own children to their patients. They reported that they always left the final decision on taking part in the NIP up to the parents.

Apart from the religious background of the HCPs, their attitude to religious objections to vaccination seemed to be important. Some of the HCPs lacked affinity with the dilemmas of the orthodox Protestant parents while others tried to understand their’ position.

Finally some HCPs reported that they were willing to discuss the vaccination decision-making process but felt they lacked the skills to do so.

### Authoritarian stance

The third manner of responding to religious objections to vaccination, described by the HCPs, was to adopt an authoritarian stance and tell the parents what they must do in their child’s best interest. In cases of tetanus post-exposure prophylaxis almost all of the GPs reported to use their medical authority to make parents comply with the immunization regimen prescribed for unvaccinated individuals.

Respondent 17, GP

Tetanus is something that you would not wish upon your worst enemy. If your kid should come down with this, you would never forgive yourself. So I say: “The wound will be cleaned and now a shot because you’ve never been vaccinated and you’ve got dirt in your system” and that is usually swallowed more or less without a problem.

Respondent 15, GP

I try anything I can think up to persuade them. Only once I didn’t succeed.

Respondent 16, GP

They have to take it. Well, … of course they are not obliged to it. But I explain them that the risk is really high in such a situation. And if I advise them to take a shot, they do so.

Respondent 19, GP

I sometimes say: “If you get any problems, just tell them that the doctor said that you had to take it.”

The GPs adopted this authoritarian stance only in cases of tetanus post-exposure-prophylaxis. Given that almost all of the GPs in these cases adopted an authoritarian stance, while none of them did so in any other cases, this approach seemed to be completely dependent on the child running a high risk on serious disease.

## Discussion

We identified three manners of responding to parents with religious objections to vaccination: the provision of medical information, the discussion of the vaccination decision-making process, and adoption of an authoritarian stance. The manner of responding was shown to depend on characteristics of the child, the willingness of the parents to engage in a discussion of the vaccination decision, and some personal characteristics of the HCPs themselves.

The three manners of responding to religious objections to vaccination resemble to recent models of medical decision making (in the context of the doctor-patient relationship) in which the informative, the shared decision-making, and the paternalistic approaches are distinguished [27-29]. There is, however, a major difference: while providing medical information on vaccination fits into the informative approach, and the adoption of an authoritarian stance on tetanus post-exposure prophylaxis fits into the paternalistic approach, discussing the vaccination decision-making process cannot be considered as shared decision-making. Shared decision making means that patient’s preferences are taken into account in a final decision that is endorsed by both the doctor and the patient. A prerequisite for shared decision making is that the available options be medically equivalent [30]. This is questionable in cases of vaccination and simply untenable in cases of tetanus post-exposure prophylaxis where refusal has a high risk of adverse outcome. Thus, the aim of discussing the vaccination decision-making process with parents who refuse vaccination is to help them make a well-considered decision; that is not necessarily a decision endorsed by the HCP.

All HCPs primarily responded by providing medical information and correcting any misconceptions regarding vaccination. They considered the provision of medical information a key competence of HCPs, and their most important contribution to acceptance of vaccination. Orthodox Protestant youngsters, however, are more interested in religious aspects of vaccination than in medical aspects [31]. And orthodox Protestant parents predominantly use religious arguments to justify their decision on vaccination [17]. Therefore the influence of medical information on parents’ final decisions is expected to be limited, as was noticed by some HCPs in the present study.

The discussion of the decision-making process required —except from parental willingness to engage in such a discussion— some religious knowledge, a positive attitude towards parents with religious objections to vaccination, and adequate communication skills.

The religious background of HCPs influences their attention to religious considerations in general clinical practice. In a recent study in the USA, older pediatricians with Christian backgrounds paid more attention to religious considerations than younger, non-religious pediatricians [32]. Similarly, GPs with a Protestant background in the Netherlands have been found to pay more attention to religious considerations in their practice than GPs with a Catholic background [33]. While to our knowledge the present study is the first to focus on the influence of religious background on vaccination discussions, our finding that in particular the HCPs with an (orthodox) Protestant background discussed the decision-making process and the religious considerations involved are in line with these studies. Some extra education on religious aspects of vaccination and training in communication skills could for the other HCPs possibly facilitate the discussion of the decision-making process with orthodox Protestant parents, however the effects of such discussions should be evaluated.

### Limitations

The data for this study were collected via interviews with HCPs, and — by definition — subjective. However, the findings are in line with the results of previous studies among orthodox Protestants [17,31]. Because of the sensitive character of the subject vaccination among orthodox Protestants, observation of the HCPs during real life consultations was not feasible. In other research the response of HCPs to simulation patients presenting with standardized problem scenarios was observed [20,21]. Although this method seems to be more objective, in these studies the trust between patient and HCP could not be taken into account. We found that orthodox Protestant parents sometimes preferred to consult their orthodox Protestant GPs with doubts about vaccination, instead of the CHC doctors and nurses who provide the vaccinations. This stresses the importance of trust. Perfectly provided medical information is not enough to make orthodox Protestant parents change their minds.

Another possible limitation on the present study is that the gender distribution of the participants was not balanced. All of the CHC doctors and nurses were female and all of the GPs were male. However, 94% of CHC doctors and the vast majority of CHC nurses in the Netherlands are female while only a third of the GPs in the Netherlands are female [34,35]. Our study population is therefore fairly representative. We tried to include female GPs in our study but, when approached, the female GPs referred us to male colleagues as they saw the orthodox Protestant patients. Given that perceived similarity of values between doctor and patient is an important factor in patient satisfaction [36], it is not surprising that orthodox protestant patients — who generally have conservative views with regard to gender roles and expect women to stay at home and care for the children [37] — tend to choose a male GP, if possible with their own religious background. The adoption of an authoritarian stance in cases of tetanus post-exposure prophylaxis, which was only seen among the GPs and thus the male participants in this study, could thus be a gender effect. This seems unlikely in light of the extenuating circumstances created by tetanus exposure, however.

## Conclusions

In this study, we identified three manners in which HCPs respond to parents with religious objections to vaccination: provision of medical information, discussion of the vaccination decision-making process, and adoption of an authoritarian stance. The choice of approach depends on the medical condition of the child, the willingness of the parents to engage in discussion, and the personal characteristics of the HCPs themselves. Given that for parents with religious objections to vaccination medical information is generally not decisive, we recommend HCPs to discuss the vaccination decision-making process – if parents are willing to engage in such a discussion- rather than to provide them with extra medical information.

## Competing interests

The authors declare that they have no competing interests.

## Authors’ contributions

WLMR conceived of the study, participated in the design, collected data, participated in analyses and drafted the manuscript. JLAH participated in the design of the study and helped to draft the manuscript. GIJ collected data and revised the manuscript, WJCvA participated in analyses and revised the manuscript, GE helped to interpret the results and revised the manuscript, KvdV participated in the design of the study and revised the manuscript. MEJL participated in the design of the study and helped to draft the manuscript. All authors read and approved the final manuscript.

## Authors’ information

WLMR is preparing a thesis on “Acceptance of vaccination among orthodox Protestants in The Netherlands.”

Further research is focusing on vaccination coverage within the various orthodox Protestant denominations, individual decisions of members of these denominations whether or not to vaccinate one’s children and the role of religious leaders in decision-making on vaccination.

## Pre-publication history

The pre-publication history for this paper can be accessed here:

http://www.biomedcentral.com/1472-6963/12/231/prepub
